# Gambling rarely stands alone: clustered addictive behaviours and socioeconomic vulnerability in adolescents

**DOI:** 10.3389/fpsyt.2026.1788018

**Published:** 2026-07-24

**Authors:** Gonzalo Romero-Martín, Eva María Andrés Esteban, Ana Ordóñez López, Carlos López Crespo, Raúl Juárez-Vela, Bárbara Oliván-Blázquez, Ana Gastón Faci, Carmen Vicente-García, Silvia Wobbeking Sánchez

**Affiliations:** 1 Centro de Enseñanza Universitaria SEK, Villanueva de la Cañada, Madrid, Spain; 2 International University of La Rioja, Logroño, Spain; 3 Faculty of Economics and Business, University Rey Juan Carlos, Madrid, Spain; 4 The Research Network on Preventive Activities and Health Promotion (redIAPP), Carlos III Health Institute, Madrid, Spain; 5 University of La Rioja, Logroño, Spain; 6 GIIS011. IIS Aragón (Instituto de Investigación Sanitaria Aragón - Institute for Health Research Aragón), Logroño, Spain; 7 Department of Psychology and Sociology, University of Zaragoza, Zaragoza, Spain; 8 Research Network of Chronicity, Primary Care and Health Promotion (RICAPPS), Zaragoza, Spain; 9 Utebo Health Centre, Aragonese Health Service, Zaragoza, Spain

**Keywords:** addiction, alcohol, drugs, gambling, teenagers, tobacco

## Abstract

**Background:**

Recent European studies estimate that between 15% and 80% of adolescents have engaged in gambling, reflecting substantial heterogeneity in measurement approaches and highlighting limitations in current surveillance systems. This variability may obscure the magnitude and urgency of the problem. Evidence suggests that adolescents exposed to socioeconomic vulnerability are more likely to engage in gambling and other co-occurring risk behaviours. This study aims to analyse gambling initiation patterns and their relationship with addictive behaviours within a socioeconomic framework.

**Methods:**

This cross-sectional observational study included 2,407 adolescents and young adults aged 14–21 years from the Autonomous Community of Madrid. Schools were selected using simple random sampling from an official registry, and approximately 95% of eligible students participated. Data were collected using a self-administered questionnaire with adequate psychometric properties (KMO = 0.843; Cronbach’s alpha = 0.861). Group differences were assessed using chisquare and Mann–Whitney U tests, and multivariate associations were examined using logistic regression models (odds ratios with 95% confidence intervals).

**Results:**

Overall, 8.6% of participants reported gambling behaviour. Gamblers were significantly older (median 18 vs 16 years, p < 0.001), more likely to be employed (OR = 2.68, 95% CI: 1.49–4.84, p = 0.001), and more likely to have repeated a school year (OR = 4.66, 95% CI: 3.07–7.06, p < 0.001). Gambling was also significantly associated with alcohol, tobacco, and illicit substance use (all p <0.001), as well as indicators of socioeconomic vulnerability.

**Conclusions:**

Adolescent gambling appears to be part of a broader cluster of socioeconomic disadvantage and co-occurring addictive behaviours rather than an isolated activity. These findings highlight the importance of integrated prevention strategies targeting multiple risk behaviours in vulnerable youth populations.

## Introduction

According to the World Health Organization (WHO), adolescence is a transitional period marked by significant physical, psychological, and social changes (1). This stage is also associated with an increased likelihood of engaging in risky behaviours, including alcohol consumption, substance use, and delinquency (2). Gambling with money is increasingly recognised as another form of risk behaviour during adolescence. These behaviours often co-occur, forming interconnected patterns that may reinforce each other and increase vulnerability to negative outcomes in both adolescence and adulthood (2, 3). Previous research has shown that individuals who engage in gambling are more likely to smoke and consume alcohol at hazardous levels (3, 4), highlighting the importance of understanding gambling within a broader constellation of risk behaviours.

Excessive gambling is considered a behavioural addiction and has gained increasing attention as a public health concern in recent years (4, 5). In Spain, regulatory frameworks have struggled to keep pace with the rapid evolution of gambling environments, particularly in relation to underage gambling. Although policies such as Royal Decree 958/2020 impose restrictions on minors’ access to gambling establishments, these measures are less effective in addressing the growing prevalence of online gambling, where most adolescent exposure now occurs.

At the European level, gambling is generally prohibited for minors (6). However, adolescents continue to engage in gambling activities despite these restrictions (7–9). This persistence is partly explained by increased exposure to gambling-related marketing, including advertising during sports events and integration within digital platforms and online games (10). Furthermore, gambling is often socially normalised as a form of entertainment in many contexts (11–13), which may further reduce perceived risk amongst young people.

Early engagement in gambling has been associated with unhealthy lifestyles, including alcohol consumption, tobacco use, and illicit substance use (12). These behaviours are shaped by a combination of individual, familial, and social factors. While the family environment plays a key role, peer influence is particularly relevant during adolescence, as social interactions strongly shape behavioural patterns related to nightlife, substance use, and gambling (14–18).

Recent research has increasingly emphasised that gambling does not occur in isolation but rather as part of a broader pattern of co-occurring behaviours embedded within specific socioeconomic contexts (19–22). Evidence shows that individuals engaging in gambling are more likely to present multiple risk behaviours simultaneously, and that these behaviours tend to cluster within certain sociodemographic profiles. In particular, socioeconomic disadvantage has been consistently associated with higher exposure to unhealthy consumption patterns, including alcohol, tobacco, and gambling (23–25).

The expansion of online gambling markets has further transformed the risk environment for adolescents. Increased accessibility, intensified advertising, and the digitalisation of gambling platforms have amplified exposure amongst young populations (22, 26–28). These structural changes suggest that gambling behaviours should be understood within a broader ecological context, where digital availability and socioeconomic conditions jointly shape behavioural patterns.

Despite this growing body of literature, important gaps remain. First, existing studies often analyse gambling or substance use in isolation, without integrating multiple risk behaviours within a single analytical framework. Second, relatively few studies explicitly examine the role of socioeconomic vulnerability in shaping gambling behaviour alongside other addictive behaviours in adolescent populations. Third, there is a lack of empirical research combining behavioural, socioeconomic, and contextual variables to provide a comprehensive understanding of how these factors interact. Addressing these gaps is essential to better understand adolescent gambling as a socially patterned phenomenon rather than an isolated behaviour.

Against this background, the present study aims to analyse gambling initiation amongst adolescents and young adults within a broader framework of socioeconomic vulnerability and co-occurring risk behaviours. Specifically, the objectives are: (1) to examine patterns of exposure to gambling involving monetary cost; (2) to identify differences between gamblers and non-gamblers according to demographic, economic, and family-related characteristics; and (3) to explore the relationship between gambling participation and other addictive behaviours, including alcohol, tobacco, internet use, and illicit substances. By situating gambling within a broader socioeconomic and behavioural context, this study seeks to contribute to a more comprehensive understanding of adolescent gambling as a socially patterned phenomenon with important public health implications.

## Material and methods

### Study

This is a cross-sectional observational study. Although initially conceptualised within a cohort framework, no longitudinal follow-up data were collected; therefore, the present analysis is strictly cross-sectional and does not allow for causal inference or temporal assessment of associations.

### Population and sample

Young people between the ages of 14 and 21 in the Autonomous Community of Madrid have been considered. It includes pre-university academic levels, from 4th year of secondary school (minimum age 14) to 2nd year of high school (where, although the maximum age should be 19, we find students repeating and therefore the age limit has been increased to 21).

The sample size was calculated using the formula for a descriptive observational design, $n=\frac{Z^{2}p(1-p)}{E^{2}}$; shere Z is the z-value according to the confidence level; p = expected proportion (since we do not know it *a priori*, we use 0.5 to maximise the sample size) and E is the precision of the estimates.

Assuming a confidence level of 95% and an accuracy for estimates of 2%, the optimal sample size would be 2,401. This number of young people will be recruited from a random sample of public schools in the Autonomous Community of Madrid. To obtain the sample size necessary to carry out the study, we used the lists of public and charter schools in the Community of Madrid. Twenty schools were selected at random, as each classroom has a maximum capacity of 25 people and there are usually between 20 and 25 students per class. Considering a minimum of two classes per academic level (4th year of secondary school, 1st and 2nd year of high school) in each school, 20 schools were sufficient. However, five reserve schools were also selected in case any of the selected schools declined to participate in the study. To obtain the necessary information, we contacted the school principals and provide them with a questionnaire that we have created ourselves.

The random selection of schools was performed using a simple random sampling procedure based on the official registry of public and charter schools in the Autonomous Community of Madrid. Each school was assigned a numerical identifier and selected using a computer-generated random number sequence. Of the initially selected schools, 100% agreed to participate, whilst the remaining declined and were replaced by pre-selected reserve schools. At the participant level, all eligible students present on the day of data collection were invited to participate. The response rate amongst students was approximately 95%, with nonparticipation primarily due to absence or lack of parental consent. No formal weighting or adjustment for non-response was performed. Therefore, potential selection bias cannot be ruled out, particularly if non-participation was associated with risk behaviours or socioeconomic characteristics.

All young people who met the following inclusion criteria were selected:

1. Be enrolled from 4th year of secondary school to 2nd year of high school at the selected school.
2. Agree to participate in the study by completing the ethical control form to participate if of legal age or have the consent of their legal guardian to participate in the study if a minor.

### Procedure and data collection

Data were collected using a self-administered questionnaire distributed in paper format or completed online via Google Forms. The questionnaire included an introductory section describing the study objectives and ethical considerations.

The instrument was developed based on prior literature on adolescent risk behaviours and included domains covering demographic characteristics, socioeconomic status, gambling exposure, and substance use. The final questionnaire consisted of 30 items, including 8 sociodemographic variables and 22 items assessing behavioural dimensions.

Operational definitions were established as follows: gambling status was defined as a binary variable (gambler vs non-gambler based on reported participation in monetary gambling activities), whilst behavioural variables (e.g., internet use, substance use) were measured using frequency-based categorical scales.

### Instrument validation

A psychometric evaluation of the 22 behavioural items was conducted using exploratory factor analysis (EFA). Three factors were identified, explaining 80.68% of the total variance:

Factor 1: Internet Use and Gaming/Gambling (70.19%)
Factor 2: Consumption of Harmful Substances (22.03%)
Factor 3: Healthy Activities (9.51%)

Sampling adequacy was confirmed using the Kaiser-Meyer-Olkin (KMO) index (KMO = 0.843). Internal consistency was assessed using Cronbach’s alpha, yielding a value of 0.861 for the overall scale, and 0.883, 0.801, and 0.707 for each of the factors, respectively, indicating good reliability.

However, the questionnaire did not include standardised measures of gambling severity, age of initiation, or detailed indicators of gambling intensity, which should be considered when interpreting the results.

### Data analysis

For the description of young people, a general descriptive study of the target population will be carried out using standard statistical parameters (mean, standard deviation, median, interquartile interval (IQI), absolute and relative frequencies, etc.).

The comparison between young players and non-players will be carried out using the chi-square test when the variable is qualitative or the non-parametric MannWhitney U test when the variable is numerical. This non-parametric contrast was used because the numerical variables were not normal. The normality test used was the Shapiro-Wilks test.

Multivariate analysis was conducted using binary logistic regression. The dependent variable was gambling status (gambler vs non-gambler). Independent variables were selected based on a combination of statistical significance in bivariate analyses (p < 0.05) and theoretical relevance supported by previous literature, in order to reduce model bias and improve interpretability.

Model calibration was assessed using the Hosmer–Lemeshow test, and discrimination was evaluated using the area under the ROC curve. Results were reported as odds ratios (OR) with 95% confidence intervals.

All analyses were conducted using STATA/SE V16.0, with a significance level of 5%.

### Ethical aspect

The study will be conducted in accordance with the principles of the Declaration of Helsinki (2008 update; available on the World Medical Association website - World Medical Association website - http://www.wma.net/e/policy/b3.htm) and in accordance with the standards of good clinical practice, as described in the ICH Tripartite Harmonised Guidelines for Good Clinical Practice (1996) and in the guidelines for Good Epidemiological Practice (http://www.ieaweb.org/GEP07.htm).

Similarly, the study will comply with Organic Law 3/2018 on the Protection of Personal Data and Guarantee of Digital Rights and Regulation (EU) 2016/679 of the Parliament and of the Council of 27 April 2016 on Data Protection (GDPR).

The study has been approved to the Ethics Committee of the International University of for La Rioja.

## Results

A total of 2,407 participants were included, of whom 8.6% (n = 208) reported gambling behaviour.

Overall, gamblers differed significantly from non-gamblers across multiple demographic and socioeconomic indicators (**Table 1**). Gamblers were older (median 18 vs 16 years, p < 0.001) and showed a more vulnerable socioeconomic profile. In particular, they were more likely to have fathers who were retired (15.38% vs 5.87%) and mothers who were unemployed or houseworkers (38.46% vs 22.04%, p < 0.001). Additionally, a higher proportion of gamblers reported a below-average household economic status (7.69% vs 3.51%, p = 0.009).

**Table 1:** Characteristics of young people according to whether they are gamblers or non-gamblers.

Variable	Total (n = 2407)	Non-gamblers (n = 2199)	Gamblers (n = 208)	p-value
Gender				0.194
Men	949 (39.43%)	869 (39.52%)	80 (38.46%)	
Women	1426 (59.24%)	1298 (59.03%)	128 (61.54%)	
Other	32 (1.33%)	32 (1.46%)	0 (0.00%)	
Age, median (IQR)	16 (15–18)	16 (15–18)	18 (16–19)	<0.001
Spanish nationality				<0.001
No	96 (3.99%)	96 (4.37%)	0 (0.00%)	
Yes	2311 (96.01%)	2103 (95.63%)	208 (100.00%)	
Father’s employment status				<0.001
Work outside the home	1989 (83.22%)	1829 (83.82%)	160 (76.92%)	
Unemployed	16 (0.67%)	16 (0.73%)	0 (0.00%)	
Houseworker	16 (0.67%)	16 (0.73%)	0 (0.00%)	
Pensioner/retired	160 (6.69%)	128 (5.87%)	32 (15.38%)	
Does not know/no answer	209 (8.74%)	193 (8.85%)	16 (7.69%)	
Mother’s employment status				<0.001
Work outside the home	1652 (69.09%)	1524 (69.81%)	128 (61.54%)	
Unemployed	128 (5.35%)	96 (4.40%)	32 (15.38%)	
Houseworker	433 (18.11%)	385 (17.64%)	48 (23.08%)	
Pensioner/retired	49 (2.05%)	49 (2.24%)	0 (0.00%)	
Does not know/no answer	129 (5.40%)	129 (5.91%)	0 (0.00%)	
Perception of household economy				0.009
On average	1830 (77.05%)	1670 (77.07%)	160 (76.92%)	
Below average	92 (3.87%)	76 (3.51%)	16 (7.69%)	
Above average	453 (19.08%)	421 (19.42%)	32 (15.38%)	
Are you currently working?				<0.001
No	2231 (93.31%)	2055 (94.14%)	176 (84.62%)	
Daily	48 (2.01%)	48 (2.20%)	0 (0.00%)	
Weekends	112 (4.68%)	80 (3.66%)	32 (15.38%)	
Average score				<0.001
Fail (less than 5)	80 (3.32%)	64 (2.91%)	16 (7.69%)	
Pass (5)	209 (8.68%)	209 (9.50%)	0 (0.00%)	
Good (6)	417 (17.32%)	385 (17.51%)	32 (15.38%)	
Very good (7 or 8)	1220 (50.69%)	1092 (49.66%)	128 (61.54%)	
Excellent (9 or 10)	481 (19.98%)	449 (20.42%)	32 (15.38%)	
Have you ever repeated a course?				<0.001
No	2198 (91.32%)	2038 (92.68%)	160 (76.92%)	
1 course	177 (7.35%)	129 (5.87%)	48 (23.08%)	
2 or more courses	32 (1.33%)	32 (1.46%)	0 (0.00%)	

In terms of economic autonomy, gamblers were more likely to be employed, particularly on weekends (15.38% vs 3.66%, p < 0.001). Academically, they also showed poorer performance: 23.08% had repeated at least one school year compared to 7.33% of non-gamblers (p < 0.001).

In summary, gambling behaviour was consistently associated with older age, socioeconomic vulnerability, early access to income, and poorer academic outcomes.

**Figure 1:**
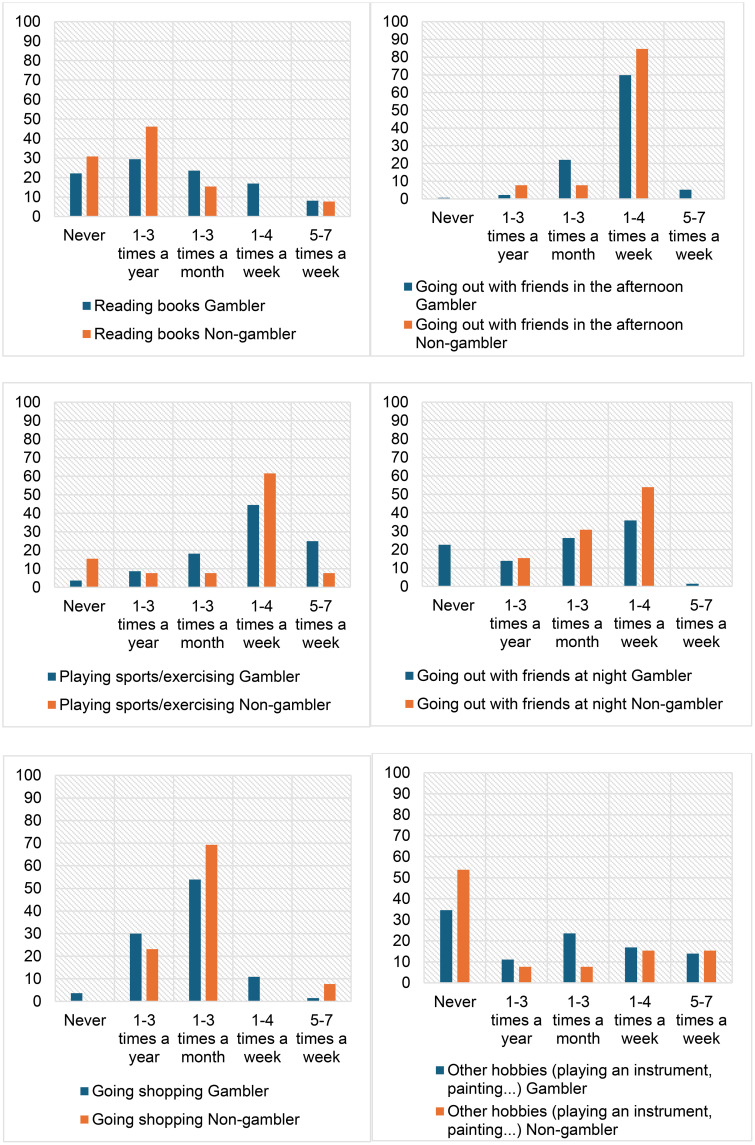
Frequency of healthy activities amongst both groups of young people.

Regarding leisure activities (**Figure 1**), gamblers reported significantly higher participation in nightlife activities. Specifically, 53.85% of gamblers engaged in nightlife activities 1–4 times per week, compared to 35.79% of non-gamblers (p < 0.001).

Similarly, gamblers showed higher engagement in online activities across all domains analysed (**Table 2**). The differences were particularly marked in higher exposure categories (15–30 hours/week and >30 hours/week), indicating more intensive use of digital platforms amongst gamblers (all p < 0.001).

**Table 2:** Frequency of online activities during young people’s free time, depending on whether they are gamblers or non-gamblers.

Activity	Category (hours/week)	Non-gamblers (n = 2199)	Gamblers (n = 208)	p-value
Instant messaging				<0.001
	Never	0 (0.00%)	16 (0.73%)	
	1–5	96 (46.15%)	914 (41.56%)	
	5–15	48 (23.08%)	866 (39.38%)	
	15–30	48 (23.08%)	339 (15.42%)	
	>30	16 (7.69%)	64 (2.91%)	
Videogames				<0.001
	Never	64 (30.77%)	370 (16.95%)	
	1–5	64 (30.77%)	980 (44.89%)	
	5–15	32 (15.38%)	497 (22.77%)	
	15–30	48 (23.08%)	272 (12.46%)	
	>30	0 (0.00%)	64 (2.93%)	
Video platforms				<0.001
	Never	16 (7.69%)	16 (0.73%)	
	1–5	64 (30.77%)	867 (39.43%)	
	5–15	48 (23.08%)	835 (37.97%)	
	15–30	80 (38.46%)	385 (17.51%)	
	>30	0 (0.00%)	96 (4.37%)	
Social media				<0.001
	Never	0 (0.00%)	129 (5.87%)	
	1–5	80 (38.46%)	803 (36.52%)	
	5–15	64 (30.77%)	817 (37.15%)	
	15–30	64 (30.77%)	386 (17.55%)	
	>30	0 (0.00%)	64 (2.91%)	
Internet use				<0.001
	Never	16 (7.69%)	114 (5.22%)	
	1–5	96 (46.15%)	1025 (46.95%)	
	5–15	48 (23.08%)	675 (30.92%)	
	15–30	16 (7.69%)	289 (13.24%)	
	>30	32 (15.38%)	80 (3.66%)	

Substance use was significantly more prevalent amongst gamblers (**Table 3**). Nondrinkers represented 30.77% of gamblers compared to 66.93% of non-gamblers (p < 0.001), indicating a substantially higher prevalence of alcohol consumption amongst gamblers. Similarly, daily tobacco use was markedly higher in gamblers (30.77% vs 5.13%, p < 0.001). Illicit substance use was also more frequent amongst gamblers (23.08% vs 12.46%, p < 0.001), with higher prevalence across most substances analysed. These findings indicate a clear clustering of gambling behaviour with substance use.

**Table 3:** Comparison of tobacco, alcohol, and illicit substance use amongst gamblers and non-gamblers.

Variable	Category	Non-gamblers (n = 2199)	Gamblers (n = 208)	p-value
Alcohol consumption				<0.001
	No, I haven’t tried it	704 (32.01%)	16 (7.69%)	
	I’ve only tried it once	768 (34.92%)	48 (23.08%)	
	Once a month or less	373 (16.96%)	64 (30.77%)	
	2–4 times a month	306 (13.92%)	64 (30.77%)	
	2–3 times a week	32 (1.46%)	0 (0.00%)	
	4 or more times a week	16 (0.73%)	16 (7.69%)	
Tobacco consumption				<0.001
	I’ve never smoked	1421 (64.64%)	64 (38.46%)	
	I’ve tried it, but I didn’t like it	438 (19.93%)	48 (7.69%)	
	I used to smoke, but I’ve quit	48 (2.20%)	0 (0.00%)	
	I smoke sporadically (<1/week)	130 (5.91%)	16 (7.69%)	
	I smoke weekly (not daily)	48 (2.20%)	16 (7.69%)	
	I smoke every day	113 (5.13%)	64 (30.77%)	
Drug consumption (any)				<0.001
	No	1925 (87.54%)	160 (76.92%)	
	Yes	274 (12.46%)	48 (23.08%)	
Type of drug used				
	Hashish or marijuana	242 (11.01%)	48 (23.08%)	<0.001
	Cocaine	0 (0.00%)	15 (7.21%)	<0.001
	Ecstasy	16 (0.73%)	0 (0.00%)	0.217
	Amphetamines or speed	0 (0.00%)	18 (8.65%)	<0.001
	Hallucinogens	0 (0.00%)	17 (8.17%)	<0.001
	Heroin	0 (0.00%)	12 (5.77%)	<0.001
	Volatile inhalants	14 (0.63%)	16 (7.69%)	<0.001
	Tranquilizers	32 (1.46%)	16 (7.69%)	<0.001

The multivariate logistic regression model (**Table 4**) identified several independent factors associated with gambling behaviour.

**Table 4:** Logistic regression model.

Variable	Category	OR	95% CI	p-value
Age	(continuous)	1.21	1.13–1.30	<0.001
Perception of household economy				
	Below average			Ref.
	On average	2.01	1.01–3.97	0.046
	Above average	1.58	0.75–3.23	0.227
Currently working				
	No			Ref.
	Yes	2.68	1.49–4.84	0.001
Father’s employment status				
	Work outside the home			Ref.
	Unemployed/houseworker 0.94		0.55–1.63	0.846
	Pensioner/retired	2.84	1.82–4.38	<0.001
Repeated a course				
	No			Ref.
	Yes	4.66	3.07–7.06	<0.001

Age was positively associated with gambling (OR = 1.21; 95% CI: 1.13–1.30; p < 0.001), indicating a 21% increase in the odds of gambling for each additional year.

The strongest association was observed for academic repetition (OR = 4.66; 95% CI: 3.07–7.06; p < 0.001), followed by having a retired/pensioner father (OR = 2.84; 95% CI: 1.82–4.38; p < 0.001) and being currently employed (OR = 2.68; 95% CI: 1.49–4.84; p = 0.001).

Perceived average household economic status was also associated with gambling (OR = 2.01; p = 0.046), although this effect was weaker and less consistent.

Overall, the multivariate model highlights academic vulnerability, early economic autonomy, and family socioeconomic characteristics as the most influential factors associated with adolescent gambling.

## Discussion

This study provides robust evidence that adolescent gambling is embedded within a broader constellation of socioeconomic vulnerability and co-occurring risk behaviours. Rather than representing an isolated activity, gambling appears to be part of a clustered behavioural profile shaped by structural, social, and individual factors. This finding aligns with previous research showing that gambling frequently co-occurs with other risky behaviours, particularly substance use (3, 4, 20), but extends the literature by explicitly integrating socioeconomic conditions into this framework.

One of the central contributions of this study is the identification of a consistent association between gambling and indicators of socioeconomic vulnerability.

Adolescents who reported gambling were more likely to come from households characterised by less favourable parental employment conditions and lower perceived economic status. This pattern is consistent with prior studies demonstrating that socioeconomic disadvantage is associated with higher levels of gambling participation and expenditure (29, 30), as well as with broader unhealthy consumption patterns (23–25). From a structural perspective, these findings suggest that gambling behaviours may be partly shaped by contextual constraints, including limited access to alternative leisure opportunities and increased exposure to environments where gambling is normalised.

In addition to socioeconomic factors, our results highlight the importance of early access to income as a potential facilitator of gambling behaviour. Adolescents who were working (particularly in precarious or informal contexts) showed significantly higher odds of gambling (OR = 2.68). This supports the hypothesis that financial autonomy at an early age may increase both opportunity and exposure to gambling activities. Previous research has suggested that access to disposable income during adolescence can influence spending behaviours and risk-taking patterns, particularly in contexts where regulatory control is limited (24, 30). Importantly, this finding underscores the need to consider not only socioeconomic disadvantage but also the mechanisms through which adolescents interact with financial resources.

Another key finding is the strong association between gambling and academic performance, particularly school repetition (OR = 4.66), which emerged as the most influential factor in the multivariate model. This result is consistent with previous evidence linking academic disengagement to higher risk of behavioural addictions and maladaptive coping strategies (31–37). From an analytical perspective, this relationship may reflect both a cause and a consequence dynamic: academic difficulties may increase vulnerability to gambling, whilst engagement in gambling and associated behaviours may further contribute to educational decline. Although causal inference cannot be established due to the cross-sectional design, the strength of this association suggests that academic performance may serve as an important marker for identifying at-risk populations.

The clustering of gambling with substance use observed in this study further reinforces the notion of shared underlying determinants. Adolescents who gamble were significantly more likely to consume alcohol, tobacco, and illicit substances, a pattern widely documented in the literature (3, 4, 20, 32). However, rather than interpreting these behaviours as independent co-occurrences, our findings support a more integrated perspective in which multiple risk behaviours are embedded within common social and environmental contexts. This interpretation is consistent with theoretical frameworks such as problem behaviour theory and socio-ecological models, which emphasise the interplay between individual characteristics and contextual factors in shaping adolescent behaviour (38).

The family context also plays a relevant role in shaping these behaviours. The higher prevalence of gambling amongst adolescents with parents in less stable employment situations suggests that household-level socioeconomic stressors may influence behavioural trajectories. This aligns with previous studies showing that family socioeconomic status is a key determinant of adolescent health behaviours and risk exposure (23–25). In this sense, gambling may function not only as a recreational activity but also as part of broader coping or adaptation strategies within constrained environments.

While previous research has often focused on individual-level predictors such as impulsivity, sensation-seeking, or gender (39, 40), the present study contributes by emphasising the role of structural and contextual determinants. This shift in perspective is particularly relevant in the context of the rapid expansion of online gambling environments, which have increased accessibility and exposure amongst adolescents (22, 26–28). The digitalisation of gambling introduces new dynamics, including constant availability, targeted advertising, and reduced supervision, which may disproportionately affect vulnerable populations.

From a public health perspective, these findings suggest that interventions targeting adolescent gambling should adopt a multidimensional approach. However, it is important to emphasise that this study does not directly evaluate the effectiveness of specific interventions or policies. Therefore, any implications for prevention strategies should be interpreted cautiously and framed as potential avenues for future research rather than definitive recommendations.

Several limitations should be considered when interpreting these findings. First, the cross-sectional design limits the ability to establish causal relationships between variables. Second, the reliance on self-reported data introduces the possibility of reporting bias, particularly under-reporting of socially undesirable behaviours such as gambling and substance use, which may lead to conservative estimates of associations. Third, the school-based sampling strategy may limit generalisability to adolescents outside the formal education system, potentially introducing selection bias. Finally, the absence of standardised measures of gambling severity and age of initiation restricts the ability to differentiate between recreational and problematic gambling.

Future research should address these limitations by incorporating longitudinal designs to examine temporal dynamics and causal pathways. In addition, the use of validated instruments for assessing gambling severity would allow for a more nuanced understanding of behavioural patterns. Further studies should also explore the interaction between socioeconomic conditions, peer networks, and digital environments in shaping adolescent gambling behaviour, particularly in the context of rapidly evolving online platforms.

## Conclusion

The findings of this study reveal a consistent and concerning pattern: adolescents and young adults from socioeconomically vulnerable backgrounds show higher exposure to gambling behaviours, particularly when early autonomy over money management, precarious employment, and adverse family economic conditions converge. This profile aligns with recent evidence within the European context, indicating that gambling initiation tends to cluster amongst youth facing structural disadvantage and reduced social protection, which also increases the likelihood of concurrent engagement in alcohol, tobacco, and illicit substance use (41–43).

By integrating demographic, socioeconomic, and behavioural variables within a single analytical framework, this study contributes to the literature by providing a more comprehensive understanding of adolescent gambling as a socially patterned phenomenon rather than an isolated behaviour. In particular, the identification of academic disengagement, early access to income, and family socioeconomic conditions as key associated factors highlights the relevance of structural and contextual determinants in shaping adolescent risk behaviours.

From a public health perspective, these findings support the importance of preventive strategies that address multiple co-occurring risk behaviours within vulnerable populations. Previous studies have highlighted the potential effectiveness of population-level approaches (such as restrictions on gambling advertising, regulation of digital environments, and limitations on the proximity of betting venues to schools) in reducing exposure and harm amongst young people (44). However, it should be noted that the present study does not directly evaluate the impact of these interventions, and such implications should therefore be interpreted with caution.

Several limitations must be considered when interpreting these results. First, the cross-sectional design precludes causal inference and limits the ability to assess temporal relationships between gambling and associated factors. Second, the use of self-reported data may introduce reporting bias, particularly underreporting of behaviours such as gambling or substance use, which could lead to conservative estimates of associations. Third, the school-based sampling strategy may limit generalisability to adolescents outside the formal education system, potentially introducing selection bias. Finally, the absence of standardised measures of gambling severity and age of initiation restricts the ability to distinguish between recreational and problematic gambling patterns.

Future research should build on these findings by incorporating longitudinal designs to better understand causal pathways and developmental trajectories. Additionally, the use of validated instruments to assess gambling severity and more detailed behavioural indicators would allow for a more precise characterisation of adolescent gambling. Further studies should also examine how socioeconomic conditions interact with peer dynamics and digital environments, particularly in the context of rapidly expanding online gambling ecosystems.

In line with recent evidence, interventions that combine regulatory approaches with educational and behavioural strategies (such as digital literacy, emotional regulation training, and harm-reduction tools) may be particularly relevant for addressing gambling and associated behaviours in vulnerable youth populations (45–48). Strengthening regulatory frameworks and adapting them to digital environments remains a key challenge for public health systems (48–53).

## Data Availability

The original contributions presented in the study are included in the article/supplementary material. Further inquiries can be directed to the corresponding authors.

## References

[1] Tozzi L Akre C Fleury-Schubert A Surís J-C . Gambling among youths in Switzerland and its association with other addictive behaviours. Swiss Med Weekly. (2013) 143:w13768. doi: 10.4414/smw.2013.13768 23572422

[2] Kock L Cox S Shahab L Roberts A Sharman S Buss V . Intersection of gambling with smoking and alcohol use in Great Britain: a cross-sectional survey in October 2022. BMJ Open. (2024) 14:e079633. doi: 10.1136/bmjopen-2023-079633 38604639 PMC11015260

[3] Grant JE Potenza MN Weinstein A Gorelick DA . Introduction to behavioral addictions. Am J Drug Alcohol Abuse. (2010) 36:233–41. doi: 10.3109/00952990.2010.491884 20560821 PMC3164585

[4] Blinn-Pike L Worthy SL Jonkman JN . Adolescent gambling: A review of an emerging field of research. J Adolesc Health. (2010) 47:223–36. doi: 10.1016/j.jadohealth.2010.05.003 20708560

[5] Adams PJ Raeburn J de Silva K . A question of balance: Prioritizing public health responses to harm from gambling. Addiction. (2009) 104:688–91. doi: 10.1111/j.1360-0443.2008.02414.x 19215607

[6] Nielsen V Blatný M Jelínek M Hrdlička M . Antisocial behavior in adolescence: Typology and relation to family context. J Early Adolescence. (2013) 33:1091–115. doi: 10.1177/0272431612445377

[7] Andrie EK Tzavara CK Tzavela E Richardson C Greydanus D Tsolia M . Gaming participation and problematic gaming correlates among European adolescents: Results from the European Addictive Behaviors Network study. Soc Psychiatry Psychiatr Epidemiol. (2019) 54:1429–41. doi: 10.1007/s00127-019-01706-w 31062040

[8] ESPAD Group . Report ESPAD 2019: Results from the European School Survey Project on Alcohol and Other Drugs. Madrid (Spain): European Monitoring Centre for Drugs and Drug Addiction (2020). Available online at: https://www.euda.europa.eu/publications/joint-publications/espad-report-2019_en (Accessed December 9, 2025).

[9] Raitasalo K Järvinen-Tassopoulos J Rask S Skogberg N . Risk and protective factors for gambling among youth by origin: Findings from three waves of the cross-sectional Finnish School Health Promotion Study among 238,939 students. J Gambling Stud. (2024) 40:1905–19. doi: 10.1007/s10899-024-10321-7 39069598 PMC11557615

[10] Armitage R . Gambling among adolescents: an emerging public health problem. Lancet Public Health. (2021) 6:e143. doi: 10.1016/S2468-2667(21)00026-8 33640074

[11] Thomas S van Schalkwyk MCI Daube M Pitt H McGee D McKee M . Protecting children and young people from contemporary gambling marketing. Health Promotion Int. (2023) 38:daac194. doi: 10.1093/heapro/daac194 36932993 PMC10024482

[12] Derevensky JL Gupta R Messerlian C Gillespie M . Youth gambling problems: the need for a responsible social policy. In: Derevensky JL Gupta R , editors.Gambling problems in young people. Madrid (Spain): Springer (2005). p. 231–52. doi: 10.1007/0-306-48586-9_12

[13] Castrén S Grainger M Lahti T Alho H Salonen AH . Risk and problems with gambling among adolescents: a convenience sample of firstyear secondary school students in Finland. Subst Abuse Treatment Prevention Policy. (2015) 10:1–9. doi: 10.1186/s13011-015-0003-8 25879923 PMC4381398

[14] Castrén S Järvinen-Tassopoulos J Raitasalo K . Money spent on gambling is associated with gambling problems: ESPAD 2019 Finland results. J Behav Addict. (2021) 10:932–40. doi: 10.1556/2006.2021.00076 34797777 PMC8987419

[15] Helmer SM Burkhart G Matias J Buck C Engling Cardoso F Vicente J . Tell me how much your friends consume: Personal, behavioral, social, and attitudinal factors associated with alcohol and cannabis consumption among European school students. Int J Environ Res Public Health. (2021) 18(4):1684. doi: 10.3390/ijerph18041684 33578655 PMC7916343

[16] Zhai ZW Yip SW Steinberg MA Wampler J Hoff RA KrishnanSarin S . Relationships between perceived family play, peer play, problematic play, and excessive alcohol consumption in adolescents. J Gambling Stud. (2017) 33:1169–85. doi: 10.1007/s10899-017-9670-x 28101835 PMC5515696

[17] Delfabbro P King DL Derevensky JL . Teenage gambling and gambling problems: prevalence, current issues, and concerns. Curr Addict Rep. (2016) 3:268–74. doi: 10.1007/s40429-016-0105-z 30311153

[18] Kristjansson AL Sigfusdottir ID Allegrante JP . Substance use among adolescents and peer use: a multilevel analysis of cross-sectional population data. Subst Abuse Treatment Prevention Policy. (2013) 8:27. doi: 10.1186/1747-597X-8-27 23902743 PMC3737037

[19] Romero-Martín G Caraballo-Pou MÁ Merchán-Hernández C . Dimensiones individual e institucional de la polarización afectiva: una propuesta de marco de análisis. Rev Española Sociología. (2024) 33:a206. doi: 10.22325/fes/res.2024.206 42004203

[20] Karaaslan KC Kesriklioğlu E . Exploring the intersection of tobacco, alcohol, and gambling for Türkiye. J Gambling Stud. (2025) 42:375–89. doi: 10.1007/s10899-025-10418-7 40728712

[21] Altıntaş M Başgül ŞS Avcu A Macit R Büyüköztürk Ş Dinçer D . A study on gambling behavior in Türkiye: Perceptions, attitudes, thoughts, and behaviors toward gambling. Psychiatry Clin Psychopharmacol. (2024) 34:311–9. doi: 10.5152/pcp.2024.24907 39772295 PMC11744376

[22] Koç Ş Kocakaya R Türkmen AS Çakıcı AB . University students’ gaming and gambling behaviors, related factors, and the relationship between gaming and gambling. J Gambling Stud. (2023) 39:1661–74. doi: 10.1007/s10899-023-10209-y 37115422

[23] Borland R Wilson N Fong GT Hammond D Cummings KM Yong H . Impact of graphic and text warnings on cigarette packs: findings from four countries over five years. Tobacco Control. (2009) 18:358–64. doi: 10.1136/tc.2008.028043 19561362 PMC4527864

[24] Gilman SE . Socioeconomic status over the life course and stages of cigarette use: initiation, regular use, and cessation. J Epidemiol Community Health. (2003) 57:802–8. doi: 10.1136/jech.57.10.802 14573586 PMC1732304

[25] Lillard DR Molloy E Sfekas A . Smoking, drinking, and other risky behaviors among American high school students. Health Econ. (2013) 22:303–22. doi: 10.1016/j.jhealeco.2012.08.006 23220458 PMC3538930

[26] Dirección General de Ordenación del Juego . Memoria anual del juego online 2024. Madrid (Spain): Ministerio de Hacienda y Función Pública, Gobierno de España (2024). Available online at: https://www.ordenacionjuego.es (Accessed December 9, 2025).

[27] European Gaming and Betting Association . Annual report 2023 (2023). Available online at: https://www.egba.eu (Accessed December 8, 2025).

[28] European Monitoring Centre for Drugs and Drug Addiction . European report on behavioural addictions. Madrid (Spain): Publications Office of the European Union (2023). Available online at: https://www.emcdda.europa.eu (Accessed December 9, 2025).

[29] Canale N Vieno A Lenzi M Griffiths MD Borraccino A Lazzeri G . Income inequality and adolescent gambling severity: Findings from a large-scale Italian representative survey. Front Psychol. (2017) 8:1318. doi: 10.3389/fpsyg.2017.01318 28824499 PMC5541014

[30] Castrén S Kontto J Alho H Salonen AH . The relationship between gambling expenditure, socio‐demographics, health‐related correlates and gambling behaviour: A cross‐sectional population‐based survey in Finland. Addiction. (2017) 113:91–106. doi: 10.1111/add.13929 28667828 PMC5763410

[31] Espadafor M Martínez S . The negative consequences of sports betting opportunities on human capital formation: Evidence from Spain. PloS One. (2021) 16:e0258857. doi: 10.1371/journal.pone.0258857 34705850 PMC8550418

[32] Lombardi G Molinaro S Cotichini R Cerrai S Scalese M Benedetti E . The cards they’re dealt: types of gambling activity, online gambling, and risk of problem gambling in European adolescents. Soc Sci Med. (2024) 363:117482. doi: 10.1016/j.socscimed.2024.117482 39536649

[33] Kristiansen S Reith G Trabjerg CM . The notorious gambling class: patterns of gambling among young people in Denmark. J Youth Stud. (2016) 20:366–81. doi: 10.1080/13676261.2016.1232480 37339054

[34] Dirección General de Ordenación del Juego . Memoria anual del juego online 2024. Madrid (Spain): Ministerio de Derechos Sociales, Consumo y Agenda 2030 (2025). Available online at: https://www.ordenacionjuego.es/novedades/memoria-anual-juego-line-ano-2024 (Accessed December 9, 2025).

[35] Grinols EL Mustard DB . Business profitability versus social profitability: evaluating industries with externalities, the case of casinos. Managerial Decision Econ. (2001) 22:143–62. doi: 10.1002/mde.1004 41531421

[36] Gruber J Köszegi B . Is addiction “rational”? Theory and evidence. Q J Econ. (2001) 116:1261–303. doi: 10.1089/glr2.2018.22106 36698420

[37] Gainsbury SM Tobias-Webb J Slonim R . Behavioral economics and gambling: A new paradigm for approaching harmminimization. Gaming Law Rev. (2018) 22:608–17. doi: 10.1089/glr2.2018.22106 36698420

[38] Jessor R . Risk behavior in adolescence: A psychosocial framework for understanding and action. J Adolescent Health. (1991) 12(8):597–605. doi: 10.1016/1054-139x(91)90007-k 1799569

[39] Oksanen A Sirola A Savolainen I Koivula A Kaakinen M Vuorinen I . Social ecological model of problem gambling: A cross-national survey study of young people in the United States, South Korea, Spain, and Finland. Int J Environ Res Public Health. (2021) 18:3220. doi: 10.3390/ijerph18063220 33804663 PMC8003601

[40] Romero Martín G . An inquiry into the dimensions, transmission channels, and applications of affective polarisation. (Tesis Doctoral Inédita). Universidad de Sevilla, Sevilla. (2024).

[41] Dowling NA Merkouris SS Greenwood CJ Oldenhof E Toumbourou JW Youssef GJ . Early risk and protective factors for problem gambling: A systematic review and meta-analysis of longitudinal studies. Clin Psychol Rev. (2017) 51:109–24. doi: 10.1016/j.cpr.2016.10.008 27855334

[42] Chóliz M Marcos M . That’s no country for ‘young’ men: A critical perspective on responsible online gambling policies for gambling disorder prevention in Spanish minors. J Gambling Issues. (2022) 49:158–73. doi: 10.4309/jgi.2022.49.7

[43] Ligneul R Sescousse G Barbalat G Domenech P Dreher J-C . Shifted risk preferences in pathological gambling. Psychol Med. (2012) 43:1059–68. doi: 10.1017/s0033291712001900 22932231

[44] Lind K Castrén S Hagfors H Salonen AH . Harm as reported by affected others: A population-based cross-sectional Finnish Gambling 2019 study. Addict Behav. (2022) 129:107263. doi: 10.1016/j.addbeh.2022.107263 35134630

[45] Håkansson A Widinghoff C . Gambling despite nationwide self-exclusion–a survey in online gamblers in Sweden. Front Psychiatry. (2020) 11:599967. doi: 10.3389/fpsyt.2020.599967 33343428 PMC7738608

[46] Gong X Zhu R . Cognitive abilities, non-cognitive skills, and gambling behaviors. J Economic Behav Organ. (2019) 165:51–69. doi: 10.1016/j.jebo.2019.06.016 38826717

[47] Cameron L Ride J Devlin N . An economic model of gambling behaviour: A two-stage approach. J Gambling Stud. (2024) 40:65–81. doi: 10.1007/s10899-022-10146-2 35867267 PMC10904484

[48] UNICEF . Children and digital marketing: Gambling and harmful products. Madrid (Spain): United Nations Children’s Fund (2023). Available online at: https://www.unicef.org (Accessed December 9, 2025).

[49] Donati MA Beccari C Sanson F Sareri GI Primi C . Parental gambling frequency and adolescent gambling: A cross-sectional path model involving adolescents and parents. PloS One. (2023) 18:e0280996. doi: 10.1371/journal.pone.0280996 36780466 PMC9925005

[50] Romero-Martín G Caraballo-Pou MÁ Merchán-Hernández C . From affective polarization to fruitful politics: A new public leadership inspired by the UN’s 2030 Agenda. Int J Public Leadership. (2023) 19:81–93. doi: 10.1108/ijpl-10-2022-0053 35579975

[51] Meisel MK Clifton AD Mackillop J Miller JD Campbell WK Goodie AS . Egocentric social network analysis of pathological gambling. Addiction. (2013) 108:584–91. doi: 10.1111/add.12014 23072641 PMC3578111

[52] Berten H Van Rossem R . Homophily in adolescence. Young. (2015) 23:76–96. doi: 10.1177/1103308814557400

[53] Li C Yu Q Zhang J Lv Z Liu Q He J . The social processes of excessive online gaming homophily: Peer selection or influence? J Youth Adolescence. (2024) 53:2393–406. doi: 10.1007/s10964-024-02032-4 38864952

